# Rat Ovarian Function Is Impaired during Experimental Autoimmune Encephalomyelitis

**DOI:** 10.3390/cells12071045

**Published:** 2023-03-30

**Authors:** Ana Milosevic, Irena Lavrnja, Danijela Savic, Katarina Milosevic, Jelena Skuljec, Ivana Bjelobaba, Marija M. Janjic

**Affiliations:** 1 Institute for Biological Research “Siniša Stanković”-National Institute of Republic of Serbia, University of Belgrade, 11000 Belgrade, Serbia; 2 Department of Neurology, University Medicine Essen, 45147 Essen, Germany; 3 Center for Translational Neuro- and Behavioral Sciences, University Hospital Essen, 45147 Essen, Germany

**Keywords:** EAE, multiple sclerosis, ovaries, steroidogenesis, progesterone, testosterone

## Abstract

Multiple sclerosis (MS) is an autoimmune disease affecting the CNS and occurring far more prevalently in women than in men. In both MS and its animal models, sex hormones play important immunomodulatory roles. We have previously shown that experimental autoimmune encephalomyelitis (EAE) affects the hypothalamic–pituitary–gonadal axis in rats of both sexes and induces an arrest in the estrous cycle in females. To investigate the gonadal status in female rats with EAE, we explored ovarian morphometric parameters, circulating and intraovarian sex steroid levels, and the expression of steroidogenic machinery components in the ovarian tissue. A prolonged state of diestrus was recorded during the peak of EAE, with maintenance of the corpora lutea, elevated intraovarian progesterone levels, and increased gene and protein expression of StAR, similar to the state of pseudopregnancy. The decrease in CYP17A1 protein expression was followed by a decrease in ovarian testosterone and estradiol levels. On the contrary, serum testosterone levels were slightly increased. With unchanged serum estradiol levels, these results point at extra-gonadal sites of sex steroid biosynthesis and catabolism as important regulators of their circulating levels. Our study suggests alterations in the function of the female reproductive system during central autoimmunity and highlights the bidirectional relationships between hormonal status and EAE.

## 1. Introduction

Multiple sclerosis (MS) is a chronic autoimmune disease of the CNS, with inflammation, demyelination, and neurodegeneration being the disease hallmarks. It affects both genders during the reproductive age. However, women are affected more frequently, with a female-to-male ratio of 2:1 or above [1]. Moreover, higher inflammatory activity in female MS patients, reflected in greater relapse frequency, is noticed only during the reproductive period [2]. 

Physiological conditions involving hormonal alterations during a woman’s life, such as puberty, menarche, pregnancy, and menopause, can impact the disease prevalence and outcome [3]. For example, the rate of relapses declines during pregnancy, especially in the third trimester, when the levels of sex hormones are at their highest. However, as sex hormone levels decline, the relapse rate increases during the first three months after childbirth and then returns to the pre-pregnancy rate [4]. A recent study showed no increased risk in the postpartum period because breastfeeding is considered a protective factor [5]. Similarly, pregnancy has protective effects in the most commonly used animal model of MS, experimental autoimmune encephalomyelitis (EAE) [6,7]. In line with the proposed protective role of sex hormones, ovariectomy induced more severe EAE features in both mice and rats [8,9]. The beneficial effects of estrogens and progesterone and its derivatives in both EAE and MS in preclinical and clinical trials have been shown, suggesting their immunomodulatory, neuroprotective, anti-inflammatory, and promyelinating effects [3,10]. Neurosteroidogenesis is impaired in both EAE [11] and MS [12], and treatment with allopregnenolone, the major progesterone-derived neurosteroid, reduces neuroinflammation and disease severity in EAE [12]. Allopregnenolone and its analogs are good candidates waiting to be tested in MS [13]. In addition, neurosteroids may have a promising effect on MS-accompanying comorbidities [14,15]. This indicates the importance of sex hormones in MS and EAE pathology, although their exact mechanisms of action remain unknown.

The main sites of sex hormones synthesis (steroidogenesis) in females are the ovaries, i.e., granulosa and theca cells of the follicles, and the corpus luteum (CL). Steroidogenic cells obtain the steroid hormones precursor, cholesterol, from de novo synthesis, hydrolysis of the stored cholesterol esters, and from circulating lipoproteins acquired from low-density lipoprotein (LDL) and high-density lipoprotein particles via the LDL receptor and scavenger receptor class B member I (SR-BI), respectively [16]. Steroidogenic acute regulatory protein (StAR) takes part in the rate-limiting step in steroidogenesis, the transfer of cholesterol from the outer to the inner mitochondrial membrane [17], where the cytochrome P450 family 11 subfamily A member 1 (CYP11A1) enzyme is located. This enzyme further converts cholesterol to pregnenolone, which, in rats, is metabolized into progesterone via the action of 3-beta-hydroxysteroid dehydrogenase (3β-HSD). By the action of 17-alpha-hydroxylase/17,20-lyase, also known as cytochrome P450 family 17 subfamily A member 1 (CYP17A1), progesterone is converted to androstenedione and then to testosterone via 17-beta-hydroxysteroid dehydrogenase activity (17β-HSD). Aromatase (cytochrome P450 family 19 subfamily A member 1, CYP19A1) further aromatizes androgens into estrogens [18,19].

Ovarian steroidogenesis is mainly regulated by pituitary gonadotropins, luteinizing and follicle-stimulating hormones (LH and FSH, respectively), secreted in response to gonadotropin-releasing hormone. Cyclic changes in gonadotropin secretion during the estrous cycle determine the expression and presence of gonadotropin receptors in the ovary. According to the “two-cell, two-gonadotropin” theory, granulosa cells express the FSH receptor (FSHR), and by its activation, FSH stimulates CYP19A1 activity and estrogen synthesis, with no effect on androgen synthesis. Theca cells express the LH receptor (LHR) through which LH stimulates androgen synthesis, without affecting CYP19A1 activity [20]. FSHR is also present in very young CL and disappears during complete luteinization. Besides theca cells, LH also binds to granulosa cells of large follicles and luteal and interstitial cells [21]. While FSH binding remains relatively constant throughout the estrous cycle, LH binding changes dramatically [22,23]. In follicles, progesterone mainly serves as a substrate for estradiol production, while the rodents’ CL represents the main source of progesterone. Sex hormone production in the CL is mainly regulated by prolactin, LH, and estradiol [24].

We have previously shown that, during acute EAE, the hypothalamic–pituitary–gonadal axis in rats of both sexes is disturbed [25] and testicular steroidogenic machinery components are downregulated [26]. Here, we show the presence of a higher number of large abnormal follicles in the ovaries at the peak of acute EAE. Alterations in the expression of ovarian steroidogenic machinery components were also observed. Our results demonstrate an increase in ovarian progesterone during the disease, together with a prominent upregulation of the StAR protein expression. On the other hand, while serum testosterone levels are increased, ovarian testosterone is diminished together with the downregulation of CYP17A1. The decrease in ovarian testosterone is followed by decreased estradiol levels in the ovarian tissue, in spite of upregulated *Cyp19a1* gene expression.

## 2. Materials and Methods

### 2.1. Animals

The studies were conducted using 9–12-week-old female rats of the *Dark Agouti* (DA) strain, provided by the Experimental animals breeding facility of the Institute for Biological Research “Sinisa Stankovic”. The animals were kept in a standard laboratory environment, with a regular circadian light/dark regime at constant room temperature and humidity and with soy pellets and tap water ad libitum. Before each experiment, the animals were assigned to cages at random and housed four per cage.

### 2.2. EAE Induction

Experimental autoimmune encephalomyelitis was induced as previously described by a subcutaneous injection of encephalitogenic emulsion, prepared by mixing equal volumes of spinal cord homogenized in phosphate buffered saline (PBS, pH = 7.4) and complete Freund’s adjuvant [25]. The animals were examined daily and given grades based on the severity of EAE symptoms: 0—no symptoms, 1—tail atony, 2—unstable gait/hind limb weakness, 3—complete hind limb paralysis, 4—moribund state. Grade 4 was set as the humane endpoint of the experiment. Body weight was also measured during the examination.

When the specific phases of the disease were reached—onset (grade 1), peak (grade 3), or end (complete recovery)—the immunized animals were sacrificed, thus forming three groups of EAE animals: Onset, Peak, and End. The untreated group (Control) was sacrificed at the end of the experiment. The experiment was repeated three times. In each experiment, five or six animals were used in each experimental group. In total, 68 animals were used.

### 2.3. Estrous Cycle Assessment

Along with symptom evaluation, the rats were subjected to vaginal smear collection and estrous cycle stage assessment. Non-invasive vaginal lavage with saline was performed every day between 9:00 and 10:00 a.m. The presence and morphology of the cells in the vaginal smear were determined via light microscopy. Animals of the Control, Onset, and End groups were sacrificed on the second day of diestrus, while those from the Peak group were sacrificed in the stage of prolonged diestrus.

### 2.4. Blood and Tissue Collection

The animals were sacrificed by asphyxia in a chamber with a gradual CO_2_ concentration increase. Blood was collected by cardiac puncture, left at room temperature to coagulate, and then centrifuged at 3000× *g* for 10 min to separate the sera. Serum samples were stored at −80 °C until further use. After blood collection, the animals were perfused with 50 mL of cold saline. Ovaries were isolated and detached from oviducts. After careful removal of the surrounding adipose tissue, both ovaries were weighed. One ovary from each animal was stored in RNAlater^®^ RNA Stabilization Solution (Ambion™, Applied Biosystems by Thermo Fisher Scientific, Waltham, MA, USA) at −80 °C for RNA isolation and gene expression analysis, while the other ovary was pooled in each experimental group, frozen in liquid nitrogen, and stored at −80 °C for protein analysis. In another experiment, one ovary was snap-frozen in liquid nitrogen and preserved for steroid extraction, and the other was fixed and further processed for histological analyses.

### 2.5. Steroid Extraction

The steroid content was extracted according to Janjic et al. [27], with some modifications. The ovaries were thawed, placed in 0.2% ethylenediaminetetraacetic acid solution in PBS (weight: volume = 1:5) and homogenized individually. The homogenate samples were snap-frozen in liquid nitrogen and thawed three times. After the final thawing step, 4 volumes of diethyl ether were added to each sample, and the mixture was vortexed thoroughly and immediately frozen again in liquid nitrogen. The upper layer, containing diethyl ether with extracted steroids, was momentarily transferred to a clean test tube. This procedure was repeated twice more with three volumes of diethyl ether each time, added to the remaining homogenate after thawing. The separated diethyl ether with steroids was pooled with the previously decanted upper layer. After the evaporation of the diethyl ether, the remaining steroid powder was dissolved in 0.1% bovine serum albumin (BSA, Sigma, St. Louis, MO, USA) solution in PBS and kept at −20 °C until steroid hormone measurements.

### 2.6. Hormone Measurements

Hormone measurements were conducted using the enzyme-linked immunosorbent assay (ELISA) method. LH levels were determined using the Rodent ELISA kit (Endocrine Technologies Inc, Newark, CA, USA). Progesterone and testosterone levels were measured using ELISA kits by Cayman Chemical Company (Ann Arbor, MI, USA; Cat. No. 582601 and 582701, respectively). Estradiol levels were determined using the 17β-Estradiol ELISA Kit (Cat. No. ADI-900-174) by Enzo Life Sciences Inc. (Farmingdale, NY, USA). For all ELISA measurements, the manufacturer’s instructions were followed in detail. After optimizing the method for each ELISA kit, the samples were adequately diluted. The results are expressed as the hormone concentration per mL for serum samples, while the hormone concentrations in extracted ovarian steroid samples were calculated relative to the weight of the starting tissue material and expressed per gram of tissue. The results are presented as the median hormone concentration with the interquartile range for each experimental group.

### 2.7. Isolation of RNA, Reverse Transcription, and qPCR

Messenger RNA from ovarian tissue was isolated using the RNeasy Mini Kit (QIAGEN, Venlo, Netherlands), following the manufacturer’s instructions. The concentration and quality of RNA as well as reverse transcription were performed as previously described [25]. The cDNA samples were used for qPCR, which was carried out using SYBR™ Green or Taqman chemistry in the QuantStudio™ 3 Real-Time PCR System (all from Applied Biosystems by Thermo Fisher Scientific). The primer pairs are shown in Table 1. The probes used for Taqman analyses were *Actb* (Rn_00667869_m1) and *Ldlr* (Rn_00598442_m1). The expression levels of target genes were quantified by the comparative 2^−∆Ct^ method, using β-actin (*Actb*) as a reference gene.

### 2.8. Protein Isolation and Western Blot

Total ovarian proteins were isolated using RIPA buffer (Sigma, St. Louis, MO, USA) containing a cocktail of protease and phosphatase inhibitors (Thermo Fisher, Rockford, IL, USA). The samples were loaded onto 12% SDS polyacrylamide gels (20 µg of protein per lane) and separated via gel electrophoresis. Western blot analyses were performed as previously described in our studies [26]. All antibodies used are listed in Table 2. A chemiluminescent reaction was induced using luminol with H_2_O_2_ as a substrate, and the signal was visualized using the iBright CL1500 Imaging System (Thermo Fisher Scientific, Rockford, IL, USA). Densitometric analysis of the blot images was performed via the ImageJ software package. The optical density (OD) of the bands of target proteins was compared to the OD of either β-actin (for StAR and CYP11A1) or total proteins (for 3β-HSD) from the same lane. The mean value of the calculated relative ODs of the Control group was set as 100% (± SEM) for each target protein (from *n* ≥ 5 Western blot analyses). The relative ODs of the Onset, Peak, and End groups were then calculated as a change in the Control values and presented as the mean change (%) ± SEM.

### 2.9. Histological Staining and Immunofluorescent Labeling of Ovarian Tissue

Ovaries isolated from the Control and Peak groups were fixed in Bouin’s solution or in 4% formalin. After dehydration, the tissue was embedded in paraffin and cut on a microtome in 7 µm-thick sections.

Throughout the ovary, sections (350 µm apart) were used for hematoxylin-eosin staining and the counting of large follicles and CL. Follicles were classified according to Pedersen and Peters [29]. Only large follicles were counted (type 5b and larger). Follicles were considered atretic if the number of pycnotic nuclei exceeded 5% of the cells. Follicles were classified as cystic based on criteria given by Lara et al. [30]. CL were classified as CL from the present and CL from the previous cycles (presence of fibrous tissue formation and necrosis), according to criteria given by Taketa [31] and Sato et al. [32]. Only large CL (above 800 µm in diameter) were counted. Ovaries of five animals per group (Control and Peak) were analyzed.

For immunofluorescent labeling, the ovarian tissue sections were deparaffinized and rehydrated. Non-specific binding was blocked with 3% BSA in PBS for 45 min at room temperature. The sections were then incubated with the primary antibody at 4 °C overnight. After washing, the sections were incubated with the secondary antibody. The information on the antibodies used is given in Table 2. Negative controls were performed by omitting the primary antibody in the incubation solution, and this resulted in no specific staining. The slides were mounted with Mowiol (Merck Millipore, Darmstadt, Germany).

Immunofluorescent labeling was examined and photographed using a Zeiss Axiovert microscope (Carl Zeiss GmbH, Vienna, Austria), and the micrographs were edited and arranged in Photoshop CC (Version 14.2, Adobe Systems Inc., San Jose, CA, USA).

### 2.10. Statistical Analysis

Statistical analyses were conducted using GraphPad Prism 8 (version 8.0.2 for Windows, GraphPad Software, La Jolla, CA, USA). The Kruskal–Wallis test followed by the uncorrected Dunn’s post hoc test was used for the analyses of multiple groups, and individual comparisons were made between the Control group and each of the EAE groups, unless indicated otherwise. The comparison between the Control and Peak groups for morphometric analyses was performed using Student’s *t*-test. The results are presented either as the mean ± SEM or as the median with the 25% percentile (quartile 1) and 75% percentile (quartile 3) for each group of animals. Statistically significant differences are labeled as: * *p* < 0.05, ** *p* < 0.01, *** *p* < 0.001, and **** *p* < 0.0001. The graphs were made using GraphPad Prism 8.

## 3. Results

### 3.1. The Monophasic Course of EAE Is Followed by a Disturbance of the Estrous Cycle

**Figure 1:**
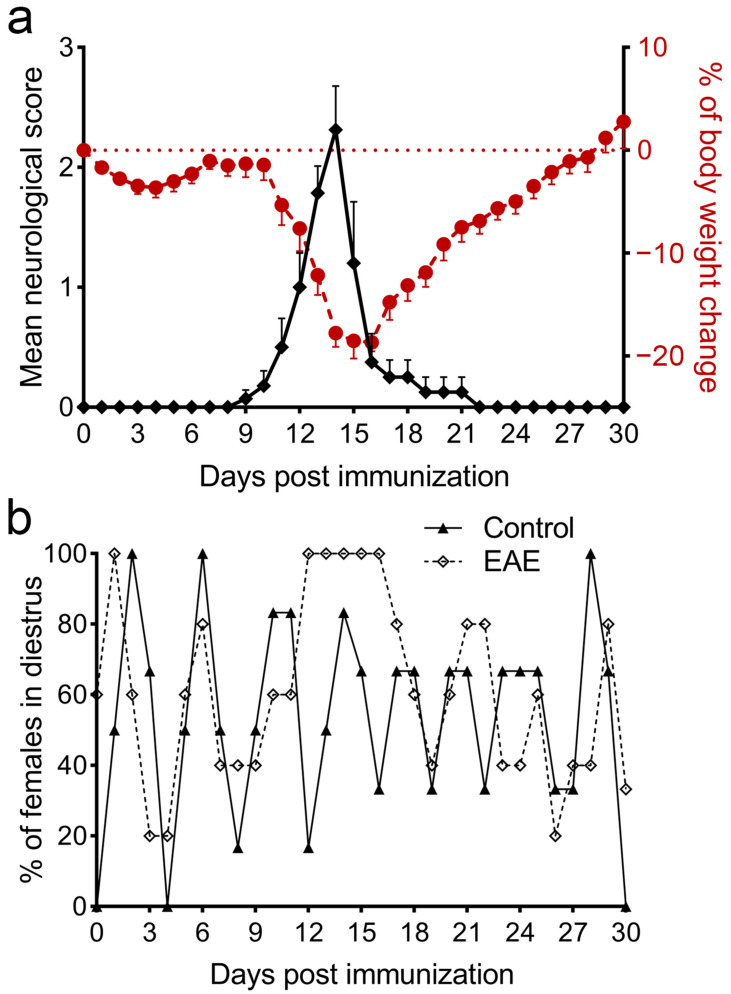
EAE course and estrous cycle dynamics in female DA rats. Neurological score, body weight, and estrous cycle were monitored for 30 dpi. (**a**) Mean neurological score (± SEM; left Y axis, black diamonds) and mean change in body weight (± SEM; right Y axis, red circles) for every dpi from a representative experiment; (**b**) The mean percentage of females in diestrus for every dpi from the Control (*n* = 6, black triangles, full line) and EAE groups (*n* = 5, open diamonds, dashed line) in a representative experiment.

A monophasic course of EAE was concomitant with body weight decline (Figure 1a), similar to our previous study [25]. The first symptoms of the disease were noted 9 days post-immunization (dpi), while the peak of EAE was reached at 14 dpi (mean score 2.31 ± 0.36); a complete absence of symptoms was registered at 22 dpi.

In contrast to the Control group, an arrest in the diestrus phase of the estrous cycle was recorded in animals with EAE, starting with the onset of symptoms and lasting 8.20 ± 0.84 days (*n* = 5). As the symptoms declined to full or partial tail atony, proestrus was noted again, and the animals continued to cycle regularly until the end of the experiment (Figure 1b).

The absolute weight of ovaries was significantly decreased at the peak of the disease; however, when normalized to the body weight measured on the same day, the results showed no differences between the groups (Table 3).

### 3.2. Ovarian Morphology in the State of Prolonged Diestrus

We have previously shown that the ovaries of females at the peak of the disease show maintenance of CL and contain all types of follicles [25].

**Figure 2:**
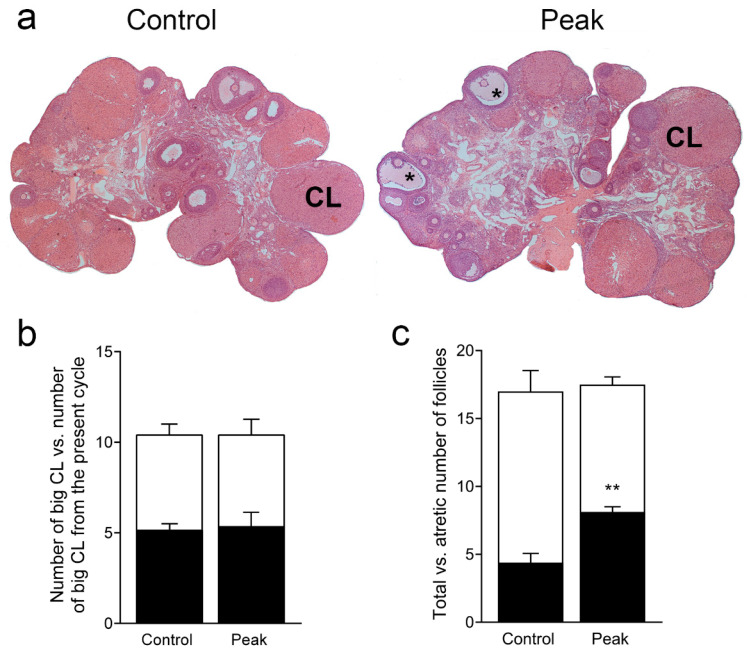
Ovarian morphology and the numbers of corpora lutea and follicles. Paraffin-embedded ovaries were cut and stained with hematoxylin-eosin. Morphometric parameters were analyzed. (**a**) Representative images of ovaries from the Control (left) and Peak (right) groups. CL—corpus luteum, *—atretic follicle; (**b**) Number of big (over 800 µm) corpora lutea (white bars) vs. number of big corpora lutea originating from the present cycle (black bars); (**c**) Total number of large follicles (white bars) vs. the number of large atretic follicles (black bars). The results (**b**,**c**) are presented as the mean number (± SEM) of CL or follicles counted from slices 350 µm apart throughout the whole ovary (*n* = 5 animals/group). ** *p* < 0.01; Student’s *t*-test.

We further characterized the morphometric parameters of the ovaries at the peak of EAE. As noted previously, large CL are observed in both control and EAE animals. In both groups, large CL (over 800 µm in diameter) with signs of cytoplasmic vacuolation of lutein cells and fibrosis were observed, implying that these large CL originated in the previous cycle, as in the Wistar Hannover strain of rats [32]. In both groups, large CL without obvious signs of degeneration were also present, suggesting that they developed in the present cycle (Figure 2a). There was no difference in the total number of large CL from the previous cycle or the number of CL from the present cycle between the analyzed groups (Figure 2b).

In both groups, all types of follicles, except for preovulatory follicles, were observed. There were no differences in the number of large follicles; however, the number of large abnormal follicles (atretic and cystic) was increased in the ovaries of animals at the peak of EAE (Figure 2c).

### 3.3. Ovarian Progesterone and Estradiol Levels and Ovarian and Serum Testosterone Levels

**Figure 3:**
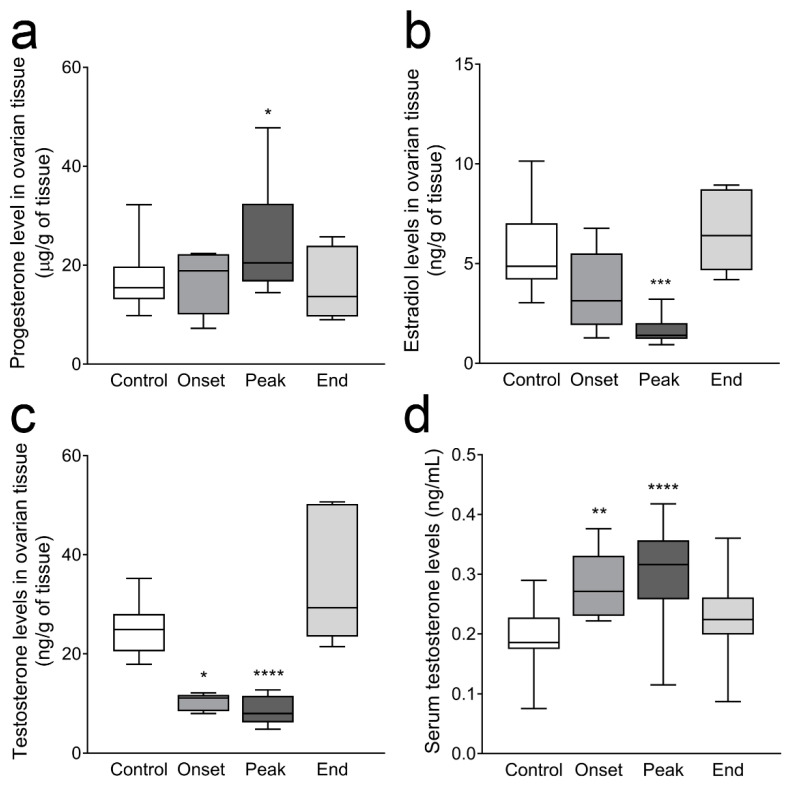
Sex steroid hormone levels in ovarian steroid extracts and in serum. Progesterone, estradiol, and testosterone levels in steroid extracts and in serum were measured by ELISA. (**a**) Ovarian progesterone (µg/g of tissue); (**b**) ovarian estradiol (ng/g of tissue); (**c**) ovarian testosterone (ng/g of tissue); (**d**) serum testosterone (ng/mL). The results are plotted as the median values of the hormone concentration. The horizontal line in each box plot indicates the median value, the box limits indicate quartile 1 and quartile 3, and the vertical whisker lines represent minimum and maximum values (*n* ≥ 5 animals/group). * *p* < 0.05, ** *p* < 0.01, *** *p* < 0.001, **** *p* < 0.0001; Kruskal–Wallis test followed by uncorrected Dunn’s post hoc test.

**Figure A1:**
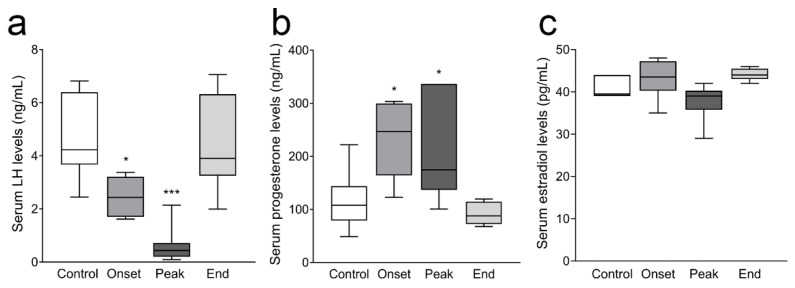
Serum levels of LH, progesterone, and estradiol. Luteinizing hormone, progesterone, and estradiol levels in serum samples were measured by ELISA. (**a**) LH (ng/mL); (**b**) progesterone (ng/mL); (**c**) estradiol (pg/mL). The results are presented as the median values of hormone concentrations. The horizontal line in each box plot indicates the median value, the box limits indicate quartile 1 and quartile 3, and the vertical whisker lines represent minimum and maximum values (*n* ≥ 5 animals/group). * *p* < 0.05, *** *p* < 0.001; Kruskal–Wallis test followed by uncorrected Dunn’s post hoc test.

We have previously demonstrated significantly decreased levels of LH in female rats, as well as elevated serum progesterone levels at the onset and peak of the disease and unchanged estradiol levels [25]. Here, we first confirmed these results (Figure A1, Appendix A). Next, we measured progesterone and estradiol levels in the steroid extracts from ovarian tissue, as well as testosterone levels in the serum and in the ovarian steroid extracts. Progesterone levels in steroid samples extracted from ovarian tissue were significantly increased at the peak of EAE (20.46 (16.72–32.42) µg/g of tissue; *p* = 0.028) compared to control levels (15.43 (13.11–19.72) µg/g of tissue; Figure 3a). In contrast to unchanged serum estradiol, the levels of this hormone, measured in ovarian steroid samples, were significantly decreased at the peak of EAE (1.40 (1.22–2.01) ng/g of tissue; *p* = 0.0002) compared to the Control values (4.86 (4.19–7.02) ng/g of tissue; Figure 3b).

Testosterone measurement in steroid samples extracted from ovarian tissue showed significantly decreased levels at the onset (11.13 (8.44–11.77) ng/g of tissue, *p* = 0.010) and peak of EAE (7.99 (6.21–11.58) ng/g of tissue, *p* < 0.0001) compared to the Control animals (24.90 (20.51–28.02) ng/g of tissue; Figure 3c). Interestingly, serum testosterone levels were increased in the Onset (0.27 (0.23–0.33) ng/mL, *p* = 0.0011) and Peak groups (0.32 (0.26–0.36) ng/mL, *p* < 0.0001) compared to the Control group of animals (0.19 (0.17–0.23) ng/mL, Figure 3d).

### 3.4. The Expression of Ovarian Enzymes and Proteins Involved in Sex Hormone Synthesis Is Affected during EAE

Next, we examined the expression of the components involved in the regulation of steroid hormone synthesis in ovarian tissue.

**Figure 4:**
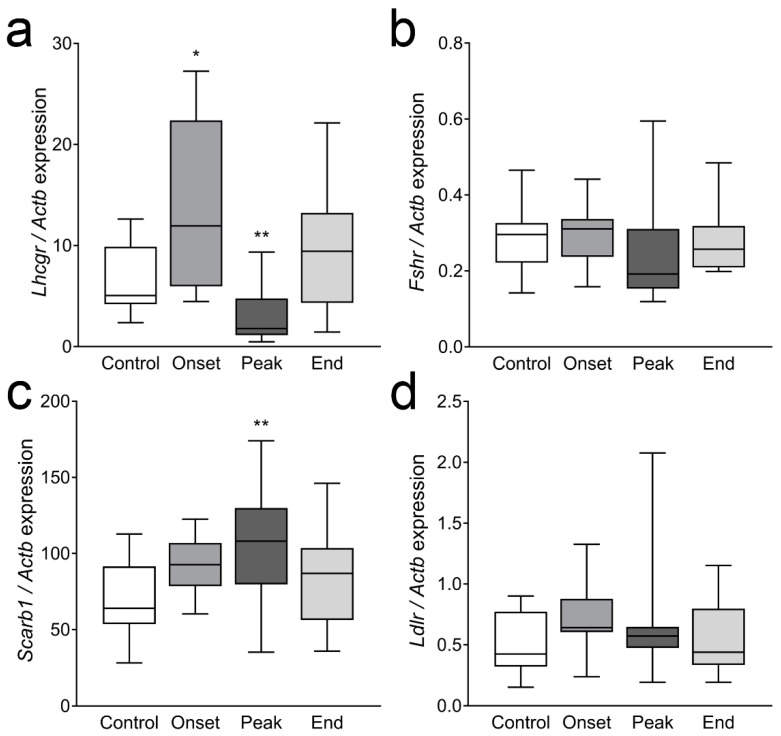
Gene expression of the receptors for gonadotropins and receptors for cholesterol uptake in ovarian tissue. Ovarian tissue from each animal of the Control, Onset, Peak, and End groups was processed for mRNA isolation, reverse transcription, and gene expression analysis by qPCR. (**a**) *Lhcgr*, (**b**) *Fshr*, (**c**) *Scarb1*, (**d**) *Ldlr*. The results are presented as the median values of relative expression of the target gene (compared to *Actb*). The horizontal line in each box plot indicates the median value, the box limits indicate quartile 1 and quartile 3, and the vertical whisker lines represent minimum and maximum values (*n* ≥ 11 animals/group). * *p* < 0.05, ** *p* < 0.01; Kruskal–Wallis test followed by uncorrected Dunn’s post hoc test.

**Figure A2:**
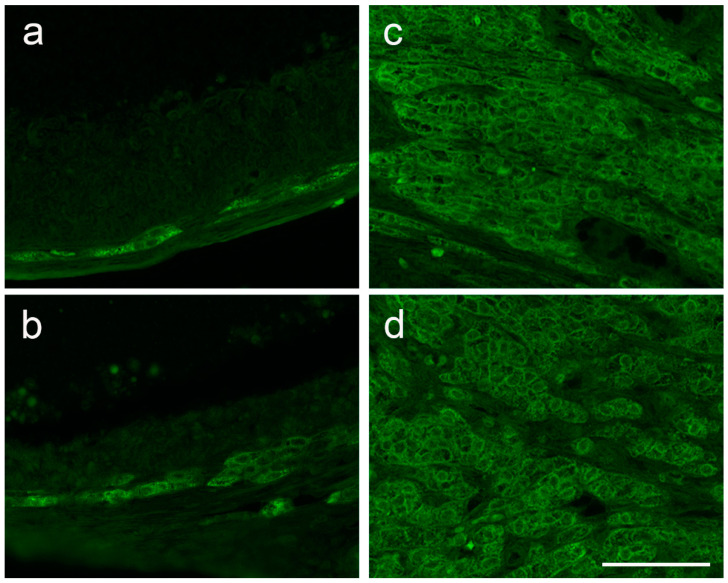
Immunofluorescent labeling of LHR in ovarian tissue. Bouin-fixed, paraffin-embedded ovaries were cut on a microtome, and the slices were labeled using an LHR antibody. (**a**) Representative follicle from the ovary of the Control group, in the diestrus phase, showing a strong LHR-specific signal in the *theca interna* region of the follicle; (**b**) Representative follicle from the ovary of the Peak group, during prolonged diestrus, showing similar LHR staining in *theca interna.* LHR staining of stroma cells was similar in the (**c**) Control and (**d**) Peak ovaries. Scale bar: 50 µm applies to all.

The gene expression of LHR and FSHR was examined. *Lhcgr* expression was upregulated at the onset of EAE (more than 2× compared to the Control, *p* = 0.03) but then decreased at the peak of the disease (about 2.5× compared to Control values; *p* = 0.0011; Figure 4a). LHR expression was also analyzed at the protein level. The antibody used was not suitable for Western blot; however, in immunohistochemistry, it produced staining of an expected distribution. No differences in LHR staining intensity or distribution could be observed throughout the disease. Representative images of the Control and Peak ovaries stained for LHR are given in Figure A2 (Appendix A). The expression of *Fshr* remained unchanged during EAE (Figure 4b).

The mRNA levels of the two receptors responsible for cholesterol uptake from circulating lipoproteins, *Scarb1* and *Ldlr*, were also examined. The expression of *Scarb1* was significantly upregulated at the peak of EAE (*p* = 0.003, Figure 4c), while *Ldlr* mRNA levels were not significantly affected (Figure 4d).

**Figure 5:**
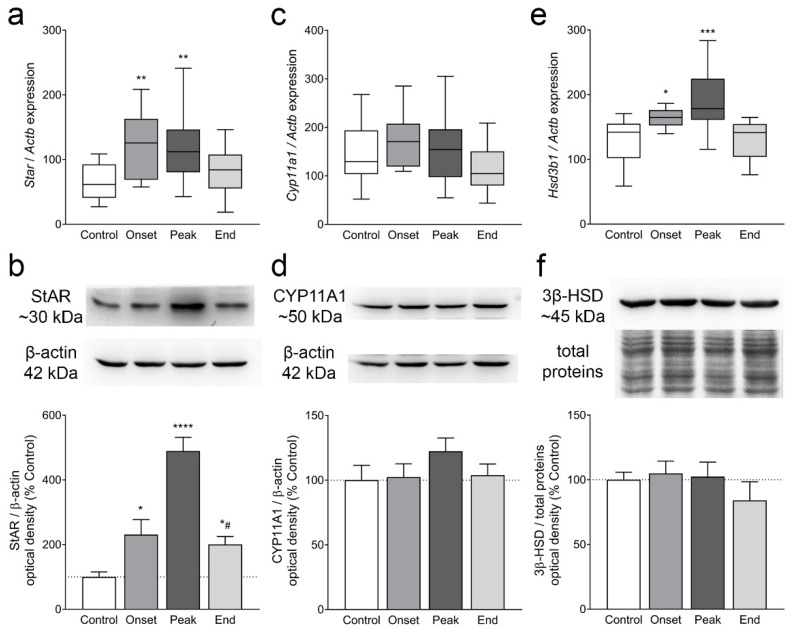
Gene and protein expression of StAR, CYP11A1, and 3β-HSD in ovarian tissue. One ovary from each animal was used for gene expression analyses by qPCR (**a**,**c**,**e**), while the other ovary was pooled for protein isolation and Western blot analysis (**b**,**d**,**f**). (**a**) *Star*; (**b**) StAR; (**c**) *Cyp11a1*; (**d**) CYP11A1; (**e**) *Hsd3b1*; (**f**) 3β-HSD. The results in (**a**,**c**,**e**) are plotted as the median values of relative expression of the target gene compared to *Actb.* The horizontal line in each box plot indicates the median value, the box limits indicate quartile 1 and quartile 3, and the vertical whisker lines represent minimum and maximum values (*n* ≥ 12 animals/group). Figures (**b**,**d**,**f**) are presented as representative blots (top panels) and quantification results (bottom panels). The results were calculated as the optical density of the target proteins relative to β-actin (**b**,**d**) or total proteins (**f**), normalized to the Control (presented as 100% ± SEM), and plotted as the mean change ± SEM (*n* ≥ 5 Western blot experiments). * *p* < 0.05, ** *p* < 0.01, *** *p* < 0.001, **** *p* < 0.0001, compared to the Control group; # *p* < 0.05, compared to the Peak group; Kruskal–Wallis test followed by uncorrected Dunn’s post hoc test.

**Figure A3:**
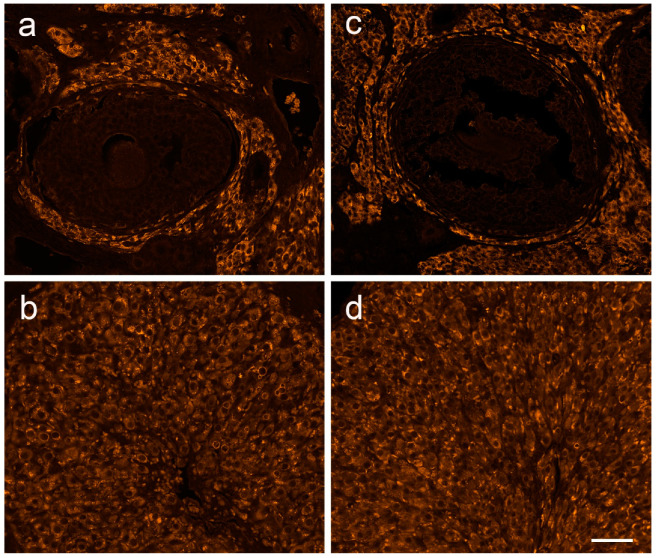
Immunofluorescent labeling of 3β-HSD in ovarian tissue. Ovaries were fixed, embedded in paraffin, and cut on a microtome. Tissue slices were fluorescently labeled using a 3β-HSD antibody. Representative images of ovaries from the Control (in the diestrus phase) and Peak (in the state of prolonged diestrus) are shown. Similar 3β-HSD-positive labeling is observed in the *theca* layer of the follicles and stroma of ovaries from the (**a**) Control and (**b**) Peak groups. Lutein cells of the CL also show a 3β-HSD-specific signal in the cytoplasm, both in ovarian sections from the (**c**) Control and (**d**) Peak, with no gross differences in the signal intensity. Scale bar: 50 µm applies to all.

Next, we analyzed the expression of StAR, CYP11A1, and 3β-HSD, involved in intracellular cholesterol processing to progesterone. The expression of *Star* was significantly upregulated at the onset (*p* = 0.005) and the peak of the disease (*p* = 0.004; Figure 5a). During the onset, a twofold increase in the StAR protein was observed (*p* = 0.047), and this increase reached a fivefold difference at the peak of the disease (*p* < 0.0001), compared to the Controls (Figure 5b). CYP11A1 mRNA and protein expression remained unchanged during EAE (Figure 5c,d). The levels of *Hsd3b1* mRNA were significantly elevated at the onset (*p* = 0.016) and the peak of the disease (*p* = 0.0003) compared to the Control group (Figure 5e), although the 3β-HSD protein levels remained unaffected (Figure 5f). Additionally, ovarian sections from the Control and Peak groups were immunofluorescently labeled for 3β-HSD protein. In both groups, 3β-HSD-specific immunopositivity was observed in the theca layer of the follicles, in stromal cells, as well as in the lutein cells of the CL. No gross differences in the distribution or signal intensity between the Control and Peak ovaries were observed in either of the regions. Representative images are shown in Figure A3 (Appendix A).

**Figure 6:**
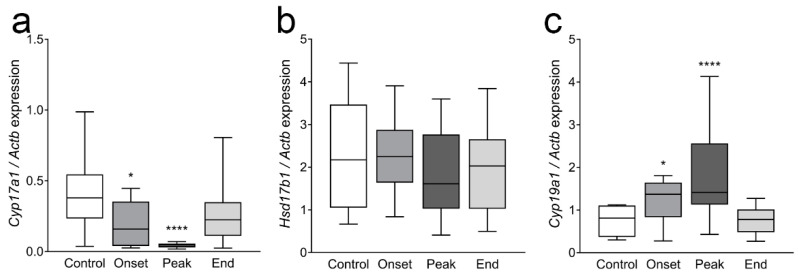
The expression pattern of *Cyp17a1*, *Hsd17b1,* and *Cyp19a1* in ovaries. One ovary from each animal of all experimental groups was processed for mRNA isolation, and gene expression was analyzed by qPCR. (**a**) *Cyp17a1*, (**b**) *Hsd17b1,* (**c**) *Cyp19a1*. The results are presented as the median values of relative expression of the target gene (compared to *Actb*). The horizontal line in each box plot indicates the median value, the box limits indicate quartile 1 and quartile 3, and the vertical whisker lines represent minimum and maximum values (*n* ≥ 12 animals/group). * *p* < 0.05, **** *p* < 0.0001; Kruskal–Wallis test followed by uncorrected Dunn’s post hoc test.

The ovarian mRNA levels of *Cyp17a1*, an androgen synthetizing enzyme, were significantly lower at the onset (*p* = 0.028) and at the peak of EAE (*p* < 0.0001; Figure 6a). The expression of *Hsd17b1*, which participates in the conversion of androstenedione to testosterone, was not affected during EAE (Figure 6b). Interestingly, the gene expression of aromatase, *Cyp19a1*, responsible for the conversion of androgens to estrogens in ovarian granulosa cells, was upregulated at the onset (*p* = 0.016) and more prominently at the peak of EAE (*p* = 0.0007; Figure 6c).

**Figure 7:**
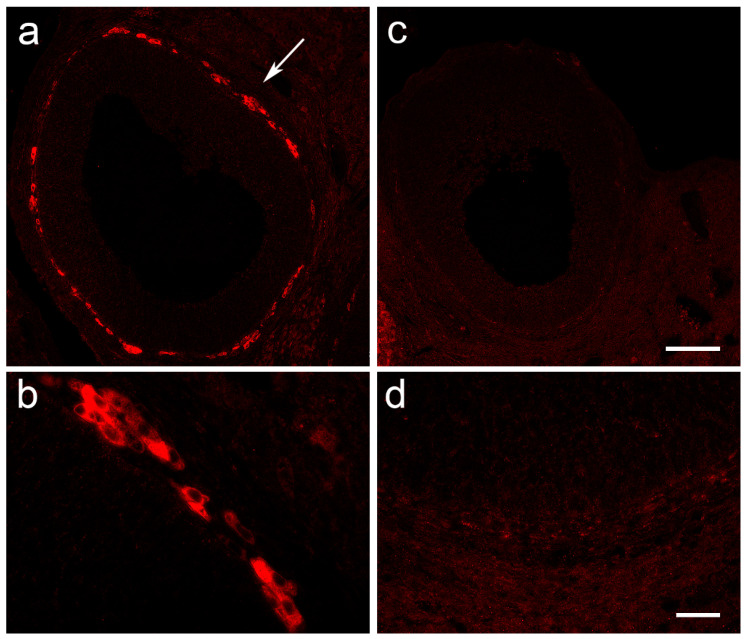
Immunofluorescent labeling of CYP17A1 in ovarian tissue. Bouin-fixed, paraffin-embedded ovaries were cut on a microtome, and the slices were labeled using CYP17A1 antibody. (**a**) Representative follicle from the ovary of the Control group, in the diestrus phase, showing a strong CYP17A1-specific signal in the *theca interna* region of the follicle (arrow). (**b**) Higher magnification of the *theca interna* region, from the Control group animal, showing CYP17A1^+^ staining in the cytoplasm of theca cells. (**c**) Ovarian tissue slice of a female from the Peak group, in prolonged diestrus lasting for four days, showing the absence of CYP17A1 labeling. (**d**) Higher magnification of ovarian tissue from the female of the Peak group. Scale bar: 100 µm—(**c**), also applies to (**a**); 20 µm—(**d**), also applies to (**b**).

Because the CYP17A1 antibody was not suitable for Western blot analysis, we performed immunofluorescent labeling in the ovarian tissue. CYP17A1-specific staining was observed in the *theca interna* layer of the follicles in the Control ovaries (Figure 7a). At higher magnification, individual *theca interna* cells were clearly labeled, excluding nuclei (Figure 7b). In the ovaries of the rats at the peak of EAE, an almost complete absence of CYP17A1 staining of *theca interna* cells was observed (Figure 7c,d). The lutein cells of both the Control and Peak rat ovaries also stained for CYP17A1, but with a lower staining intensity than that observed in the *theca interna* layer in the Control ovaries (data not shown). The recovery of CYP17A1 staining was observed at the end of the disease (data not shown).

**Figure A4:**
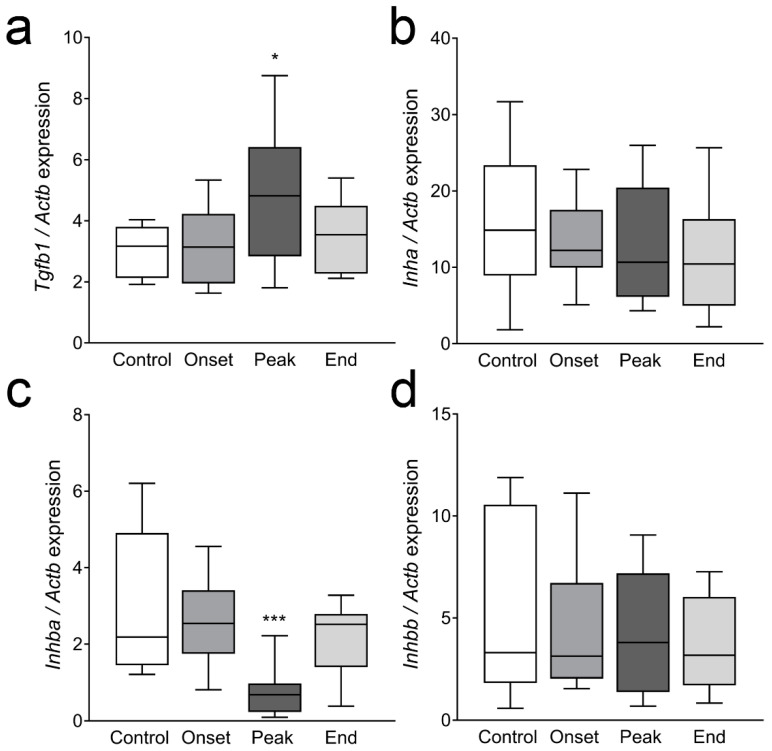
Ovarian gene expression of TGFβ family members. Ovaries from all experimental groups were isolated and processed for qPCR analysis. (**a**) *Tgfb1*; (**b**) *Inha*; (**c**) *Inhba*; (**d**) *Inhbb*. The results are plotted as the median values of relative expression of the target gene (compared to *Actb*). The horizontal line in each box plot indicates the median value, the box limits indicate quartile 1 and quartile 3, and the vertical whisker lines represent minimum and maximum values (*n* ≥ 11 animals/group). * *p* < 0.05, *** *p* < 0.001; Kruskal–Wallis test followed by uncorrected Dunn’s post hoc test.

The gene expression of transforming growth factor beta (TGFβ) family members was examined in ovarian tissue as well (Figure A4, Appendix A). The expression of *Tgfb1* was significantly upregulated only in the Peak group (*p* = 0.016; Figure A4a). The levels of mRNA of *Inha*, coding for the inhibin alpha subunit were unchanged during the disease (Figure A4b). The gene expression of the inhibin beta A subunit, *Inhba*, was significantly downregulated at the peak of EAE (*p* < 0. 001; Figure A4c), while no changes in the expression of the inhibin beta B subunit, *Inhbb*, were recorded during EAE (Figure A4d).

## 4. Discussion

The nature of the connection between ovarian function and MS is still under-explored. Lower anti-Müllerian hormone levels were recorded in women with MS [33], and this parameter, along with a lower total antral follicle count and ovarian volume, was associated with higher disease activity [34,35]. Nevertheless, a clear connection between infertility and MS is not proven [36]. In this study, we aimed to elucidate ovarian status during acute EAE, the most exploited animal model of MS.

Here, we record an arrest in the diestrus state during the symptomatic phase of EAE, which is consistent with our previous study [25]. Even though this is followed by a decrease in ovarian weight, when normalized to the body weight, this decrease is annulled. Moreover, the arrest in diestrus is not followed by a decrease in the number of big CL or the number of large follicles. Elevated levels of progesterone in the serum and in the ovaries, as well as histological analyses, imply that CL remain functional, despite the fact that rodent CL are ultrashort-lived and should regress during prolonged diestrus in EAE animals. Two conditions during which functional CL in rats are maintained and which cause an arrest in the estrous cycle are pregnancy and pseudopregnancy [37,38]. Here, we present, for the first time, that female rats during EAE are in a state similar to pseudopregnancy.

Aside from CL maintenance, pseudopregnancy is also characterized by a rise in the number of large precystic and atretic follicles [39], as observed herein at the peak of the disease. Atresia in goats was followed by decreased intraovarian estradiol levels [40], as we report here as well. We hypothesize that, like in pseudopregnancy [41], the observed increase in the number of atretic follicles comes as a consequence of LH levels that are insufficient to cause full maturation of the follicles and preovulatory estradiol release. In addition, the observed decrease in *Inhba* mRNA levels at the peak of EAE corresponds to the downregulation of *Inhba* observed during early atresia in lambs [42], while in mice, increased atresia was correlated with activin receptor type II deficiency, which can consequentially be reflected in decreased activin/inhibin signaling [43].

Here, we report increased mRNA and protein levels of StAR along with an increase in the serum progesterone concentration, all of which have already been observed during pseudopregnancy [44]. The main hormone responsible for the rescue of CL during pregnancy and pseudopregnancy in rodents is prolactin [45]. Although we did not measure prolactin levels in this study, it has already been shown that serum prolactin levels are elevated in immunized female rats [46,47] as well as in MS patients of both sexes [48]. In addition, we recorded a fluctuation in the *Lhcgr* mRNA level during EAE, an increase during the onset, and then a dramatic decrease during the peak of the disease, similar to its fluctuations in the first days of pseudopregnancy [44].

The presence of numerous large CL during EAE implies that the observed increase in progesterone levels probably results from the activity of lutein cells. Indeed, progesterone-producing steroidogenic machinery components—StAR, CYP11A1, and 3β-HSD—are highly expressed in CL, and prolactin is one of their positive regulators [24]. However, follicles may also partially contribute to progesterone secretion. Namely, we have previously shown that *Fshb* mRNA increases in the pituitary during EAE [25], implying increased secretion of FSH [49,50,51]. It is also interesting that women with MS may have elevated FSH levels during the luteal phase [52], correlating with a higher disability score [53]. FSH alone can stimulate progesterone synthesis in granulosa cells [54,55,56], and this effect is enhanced with the presence of TGFβ [57]. The upregulation of *Fshb* [25] and the upregulation of *Tgfb1* during EAE, reported here, could also be responsible for *Scarb1* induction by follicular granulosa cells [58], while prolactin may be responsible for the *Scarb1* induction in luteal cells [59]. An increase in the mRNA level of *Star* and *Hsd3b1* during the EAE onset and peak was followed by an induction of StAR protein expression only. This is in line with other studies, which reported the stimulatory effect of FSH on the StAR gene and protein expression in granulosa cells, enhanced by TGFβ [60,61]. An FSH stimulatory effect was also reported on the 3β-HSD gene and protein expression, while the reports about its effects on CYP11A1 expression in rat granulosa cells are not conclusive [61,62]. Together, our results show that a rise in serum progesterone levels corresponds to ovarian progesterone levels, indicating its ovarian origin.

It is interesting to speculate that the induction of endogenous progesterone production during EAE might contribute to the spontaneous resolution of the disease, since progesterone treatment has been shown to be immunomodulatory and neuroprotective in EAE [63]. However, this seems to be specific for the acute EAE model, because no changes in circulating progesterone levels were reported in female patients with MS [52,53,64,65].

Our results suggest that the decrease in the androgen-producing enzyme, CYP17A1 mRNA, arises due to a strong downregulation of its expression in theca cells, because it is generally assumed that CYP17A1 in rat follicles is expressed exclusively in these cells [20]. This is not surprising because this enzyme is dependent on LH, the levels of which are lower at the peak of EAE. Accordingly, this is accompanied by lower intraovarian testosterone levels.

On the other hand, our results demonstrated an increased expression of the aromatase coding gene *Cyp19a1* during EAE, which might result from the rise in FSH [25], which has been shown to induce aromatase gene expression in rat granulosa cell cultures [66]. Nevertheless, the reduction in ovarian testosterone production is followed by a reduction in the ovarian estradiol level because of the lack of a substrate for aromatase. Interestingly, lower circulating testosterone levels were recorded in women with MS [53,64], and the application of this hormone has been proven to be beneficial in both MS and EAE [67].

Further, we consistently observe no changes in the estradiol serum concentration and a small but significant rise in serum testosterone levels during EAE. The concentrations of sex steroids in the circulation are maintained through the coordinated regulation of steroid biosynthesis and elimination, and the disturbance of these processes could lead to the discrepancy between gonadal and circulating levels. Elimination represents hydroxylation by CYP enzymes and conjugation with glucuronide and sulfate, which are among the major hepatic pathways of steroid inactivation [68]. Although we did not observe gross changes in the appearance of liver tissue (data not shown), liver damage occurs in EAE [69], implying that sex-steroid catabolism may also be disturbed.

Another explanation for the discrepancy between gonadal and circulating testosterone and estradiol levels may be their increased production in other tissues. Namely, a certain amount of sex steroids in circulation stems from “non-classical” steroidogenic organs such as the brain, skin, adipose tissue, etc. [70]. For example, white adipose tissue (WAT) represents an important site for steroid hormone storage, synthesis (both de novo and from precursors), and metabolism [71,72,73], and rats’ WAT expresses genes encoding all components of the sex steroidogenic machinery [74,75]. EAE leads to a dramatic reduction in WAT in mice during EAE [76], and increased lipolysis in cows is related to higher circulating concentrations of androgens [77]. Since we also report a significant loss of body weight, this could contribute to the discrepancy between testosterone and estradiol levels in serum and ovaries in our experiments.

## 5. Conclusions

We report for the first time that the disruption of the estrous cycle during EAE is associated with changes in ovarian morphology—in particular, the prolonged maintenance of functional CL and a more pronounced process of atresia. In addition, ovarian steroidogenesis is also disrupted, contributing to an overall pseudopregnancy-like state that likely represents an adaptation to the ongoing inflammation and implies a temporary reduction in the reproductive capacity to combat the disease. Furthermore, the elevated levels of circulating progesterone, testosterone, and steady state estradiol may represent a self-defense mechanism that helps control the intensity of the inflammatory response and contribute to complete remission in the acute EAE model. Although sex hormones have been shown to be neuroprotective and promising agents in preclinical and clinical studies in MS, their use in patients is complicated by the individual hormonal status and the possible induction of other health risks, which hinders their approval as an official or adjuvant therapy. Therefore, monitoring the hormonal status in MS patients should be considered as part of a regular follow-up, which could be helpful for better managing of the disease with current treatments or developing new therapeutic approaches.

## Figures and Tables

**Table 1:** Primer pairs used for qPCR.

Primer	Sequence	Accession Number
*Actb*	forward: AGATTACTGCCCTGGCTCCT reverse: ACATCTGCTGGAAGGTGGAC	NM_031144.3
*Cyp11a1*	forward: ACTTCCTGAGGGAGAACGGC reverse: TCCATGTTGCCCAGCTTCTC	NM_017286.3
*Cyp17a1*	forward: GTTTCTCCCCAGACGTGGTC reverse: GGTCCGACAAGAGGCTTTGA	NM_012753.2
*Cyp19a1*	forward: CTCAACCTCACCACGGATGT reverse: GGCTCTCTGGATGGATGCTC	NM_017085.2
*Fshr*	forward: CTACACATTGACAGCCATCACCCTA reverse: GGCAAAAGTCCAGCCCAATACC	NM_199237.1
*Hsd17b1*	forward: ATGAGTTGAACGCTGTGGGT reverse: GGCACAGTACACTTCGTGGA	NM_012851.2
*Hsd3b1*	forward: GACAGGAGCAGGAGGGTTTGTGG reverse: CTCCTTCTAACATTGTCACCTTGGCCT	NM_001007719.3
*Inha*	forward: GCCTGAGACCACTCCTTTCC reverse: AAGGAGATGTTGAGGGCAGC	NM_012590.2
*Inhba*	forward: CTGGCAAAACAGAAGGGACC reverse: GCATCCTGGCAGCAAAAGTC	NM_017128.2
*Inhbb*	forward: TCCTAGTGCCCTGCTGAGAT reverse: ACCCACAGGGACAACTTCTG	NM_080771.1
*Lhcgr*	forward: TATGCTCGGAGGATGGCTCT reverse: AGCACAGATGACGACGAAGG	NM_012978.1
*Scarb1*	forward: GCTTCTGGTGCCCATCATTTAC reverse: AGCTTGGCTTCTTGCAGTACC	NM_031541.1
*Star*	forward: AGCAAGGAGAGGAAGCTATGC reverse: GGCACCACCTTACTTAGCACT	NM_031558.3
*Tgfb1*	forward: CTGCTGACCCCCACTGATAC reverse: AGCCCTGTATTCCGTCTCCT	NM_021578.2

**Table 2:** Antibodies used in Western blot (WB) and immunofluorescent labeling (IF).

Antibody	Source and Type	Manufacturer	Dilution
β-actin	Mouse, monoclonal	Sigma, A5316	1:5000 ^WB^
StAR	Rabbit, polyclonal	Gift from Prof. Vimal Selvaraj [28]	1:500 ^WB^
CYP11A1	Rabbit, polyclonal	Protein Tech, 13363-1-AP	1:2000 ^WB^
3β-HSD	Mouse, monoclonal	Santa Cruz, sc-515120	1:500 ^WB^ 1:50 ^IF^
LHR	Rabbit, polyclonal	Abcam, ab125214	1:200 ^IF^
CYP17A1	Rabbit, polyclonal	Protein Tech, 14447-1-AP	1:500 ^IF^
Anti-mouse IgG, HRP-conjugated	Donkey, polyclonal	Santa Cruz, sc-2314	1:5000 ^WB^
Anti-rabbit IgG, HRP-conjugated	Donkey, polyclonal	Santa Cruz, sc-2313	1:5000 ^WB^
Anti-mouse IgG, Alexa Fluor 555-conjugated	Goat, polyclonal	Life technologies, A21424	1:400 ^IF^
Anti-rabbit IgG, Alexa Fluor 555-conjugated	Goat, polyclonal	Life technologies, A21429	1:400 ^IF^
Anti-rabbit IgG, Alexa Fluor 488-conjugated	Goat, polyclonal	Invitrogen, A11008	1:400 ^IF^

**Table 3:** Absolute and relative ovarian weight.

Ovarian Weight	Control	Onset	Peak	End
Absolute (mg)	48 (41–52)	46 (42–50)	36 (27–40) *	49 (46–51)
Relative to body weight (mg/g)	0.28 (0.24–0.30)	0.30 (0.26–0.33)	0.28 (0.22–0.30)	0.29 (0.28–0.31)

The data are presented as the median (quartile 1–quartile 3) (*n* = 5 animals/group, from representative experiment). * *p* < 0.05; Kruskal–Wallis test followed by uncorrected Dunn’s post hoc test.

## Data Availability

Data presented in this study are available in the article. Raw data are available upon request from the corresponding author M.M.J.
